# Designed mono- and di-covalent inhibitors trap modeled functional motions for *Trypanosoma cruzi* proline racemase in crystallography

**DOI:** 10.1371/journal.pntd.0006853

**Published:** 2018-10-29

**Authors:** Patricia de Aguiar Amaral, Delphine Autheman, Guilherme Dias de Melo, Nicolas Gouault, Jean-François Cupif, Sophie Goyard, Patricia Dutra, Nicolas Coatnoan, Alain Cosson, Damien Monet, Frederick Saul, Ahmed Haouz, Philippe Uriac, Arnaud Blondel, Paola Minoprio

**Affiliations:** 1 Université de Rennes 1, Equipe Chimie organique et interfaces (CORINT), UMR 6226 Sciences Chimiques de Rennes, Rennes, France; 2 Institut Pasteur, Laboratoire des Processus Infectieux à Trypanosomatidés, Département Infection et Epidémiologie, Paris, France; 3 Institut Pasteur, Unité de Bioinformatique Structurale, Département de Biologie Structurale et Chimie, CNRS-UMR 3528, Paris, France; 4 Institut Pasteur, Plateforme de Cristallographie, Département de Biologie Structurale et Chimie, CNRS-UMR 3528, Paris, France; National Institute of Allergy and Infectious Diseases, UNITED STATES

## Abstract

Chagas disease, caused by *Trypanosoma cruzi*, affects millions of people in South America and no satisfactory therapy exists, especially for its life threatening chronic phase. We targeted the Proline Racemase of *T*. *cruzi*, which is present in all stages of the parasite life cycle, to discover new inhibitors against this disease. The first published crystal structures of the enzyme revealed that the catalytic site is too small to allow any relevant drug design. In previous work, to break through the chemical space afforded to virtual screening and drug design, we generated intermediate models between the open (ligand free) and closed (ligand bound) forms of the enzyme. In the present work, we co-crystallized the enzyme with the selected inhibitors and found that they were covalently bound to the catalytic cysteine residues in the active site, thus explaining why these compounds act as irreversible inhibitors. These results led us to the design of a novel, more potent specific inhibitor, NG-P27. Co-crystallization of this new inhibitor with the enzyme allowed us to confirm the predicted protein functional motions and further characterize the chemical mechanism. Hence, the catalytic Cys300 sulfur atom of the enzyme attacks the C2 carbon of the inhibitor in a coupled, regiospecific—stereospecific Michael reaction with trans-addition of a proton on the C3 carbon. Strikingly, the six different conformations of the catalytic site in the crystal structures reported in this work had key similarities to our intermediate models previously generated by inference of the protein functional motions. These crystal structures span a conformational interval covering roughly the first quarter of the opening mechanism, demonstrating the relevance of modeling approaches to break through chemical space in drug design.

## Author summary

There is an urgent need to develop innovative medicines addressing neglected diseases, multi-drug resistance and other unmet therapeutic needs. To create new drug design opportunities, we attempted to exploit protein functional motions by using a rational approach to model structural intermediates of a therapeutic target. After successfully designing inhibitors based on modeled intermediates of *T*. *Cruzi* proline racemase, the determination of crystal structures of the target protein in complex with the inhibitors revealed conformations that were strikingly close to the predicted models. Thus, beyond the discovery of compounds establishing a novel mode of action that can lead to innovative treatments of Chagas disease, we illustrate how modeling protein functional motions can be exploited in a rational approach to create opportunities in drug design.

## Introduction

Chagas disease, with nearly 10 million people infected and 100 million at risk, is the principal cause of lethality from neglected tropical diseases in Central and South America, and a leading one among all infectious diseases [1]. It is often fatal to young children and incurable in its chronic phase, which persists for decades and inflicts 10% annual lethality [2, 3]. In addition to its severe socioeconomic burden [1], Chagas disease is becoming a global concern as it extends to northern countries following human migrations.

There is currently no effective vaccine, and after substantial restriction in indications for the use of Nifurtimox due to severe side effects [4, 5], Benznidazole is the only generally available drug, but still causes serious side effects [6]. Drugs can be efficient in the acute phase, but are of questionable value in the chronic phase of the disease. Hence, it is considered a priority to find more effective treatments [7, 8]. Unfortunately, no satisfactory compounds have been identified so far [9] despite the identification of promising therapeutic targets [10].

Proline racemase of *Trypanosoma cruzi* (*Tc*PRAC) is an enzyme present at all stages of the parasite life cycle, contributing to immune escape and persistence of the parasite in the host [11, 12]. Gene over-expression increases virulence, while knockdown appears lethal for *T*. *cruzi* [13]. Interestingly, a transition analog inhibitor of *Tc*PRAC, 2-pyrrolecarboxylic acid (PYC), reduces *in vitro* infection in a dose-dependent manner [14] and decreases the mean number of parasites per cell [15]. These results, despite the poor solubility of PYC, supported *Tc*PRAC as a promising target to fight Chagas disease.

Co-crystallization of *Tc*PRAC with PYC showed highly specific and tightly closed catalytic sites of the enzyme (pdb 1W61), which left almost no space for modulation of inhibitor candidates [16]. Accordingly, conventional drug design strategies based on this structure proved unsuccessful. The structure also showed that *Tc*PRAC crystallizes as a homodimer and bears a catalytic site on each subunit. Interestingly, a crystal of a hemi-saturated form (pdb 1W62) showed the free subunit catalytic site in an open form. This suggested that the enzyme function involved an opening/closing mechanism with intermediate forms that could be used to identify and design novel types of inhibitors. To test this hypothesis, we modeled plausible intermediates of the functional opening/closing motions of *Tc*PRAC [17]. This strategy allowed an expansion of the chemical space used in our virtual screening procedure and led us to identify two inhibitors of the enzyme, (E)-4-Oxo-pent-2-enoic acid (OxoPA) [18] and its derivative (E)-5-Bromo-4-oxo-pent-2-enoic acid (BrOxoPA) which were far more potent than PYC [17].

Although conventional strategies in drug design focus on competitive inhibitors, the identification of covalent inhibitors is fortunate considering the growing interest in this type of enzymatic inhibitors [19, 20]. Indeed, a number of previously known anti-infectious agents (β-lactames, antivirals) [21, 22] or more recently the proton pump inhibitors [23], are irreversible inhibitors that have been successfully used for therapeutic purposes. This strategy was used by Ellmann and colleagues to design cruzipaine inhibitors for *T*. *cruzi* [24, 25]. Optimization of irreversible inhibitors requires the development of advanced and specific methods taking pre- and post-reaction states into account in the design of chemical chemical analogues aiming at therapeutic use [23].

Resolution of the OxoPA and BrOxoPA co-complexes in this work turned out to be a key step in the design of improved drug candidates, since it showed their detailed atomic interactions in the catalytic site and identified atoms involved in covalent bond formation. This enabled us to model the candidate structures and their interactions with the enzyme both before and after the reaction, allowing the induced changes in chemical connectivity and geometry to be taken into account in the design process. Accordingly, we could probe the determinants for affinity and selectivity by modulation of the electrophilic moieties of design candidates taking into account the position of the nucleophilic catalytic cysteine. The designed candidate displaying the best *Tc*PRAC enzymatic inhibition, NG-P27, exhibited trypanostatic/trypanocydal activity in preliminary *in vitro* experiments. The crystal structure of NG-P27 in complex with the enzyme revealed the position of the inhibitor after reaction along a regiospecific and stereospecific Michael mechanism. The structure also revealed that the cyclopentane moiety of the inhibitor could adopt multiple conformations, suggesting that space was available for further chemical modulations. Interestingly, the conformation of the active site in the co-crystal structures with our inhibitors was highly similar to that of the transitional intermediate models built to identify the first inhibitors by virtual screening [17]. This could be viewed as a demonstration of the relevance of molecular modeling in enlarging chemical space search in drug design.

## Methods

### Preparation of recombinant *Tc*PRAC

Recombinant *Trypanosoma cruzi* proline racemase (EC 5.1.1.4) was produced in *E*. *coli* BL21 (DE3) (Invitrogen) and purified by immobilized metal affinity chromatography on nickel columns, as previously described [11].

### Racemization of L-Proline and inhibition assays

Proline racemization conditions for *Tc*PRAC were determined as previously described [12] and L- to D- proline conversion took place in 1.0 mL reaction. The concentrations of D-proline were determined by optical rotation of the solution at 365 nm with a 10 cm optical path cell, at 37°C for 10^3^ seconds using a polarimeter (Jasco, P-2000, Bouguenais, France). Assays were performed as follows: 40 mM of L-Proline in 0.2 M sodium acetate pH 6.0 and 5 μg/mL of *Tc*PRAC were loaded into tubes with serial dilutions of PYC, OxoPA and BrOxoPA (from 0.3125 to 5 μM), or similar concentrations of potential optimized inhibitors.

### Kinetics analysis

Inactivation kinetics were performed at different inhibitor concentrations as described previously [17, 26, 27]. A program developed in-house was used to fit the data [17, 26, 27]. As global fitting appeared difficult and was unsuccessful, single exponential models were simply fit to the individual kinetics. Each fit was checked visually.

### Crystallization, X-ray data collection, processing, and refinement

Crystallization screening trials for *Tc*PRAC in complex with the compounds BrOxoPA, OxoPA, and NG-P27 were carried out by the sitting drop vapor-diffusion method with a Mosquito automated nanoliter dispensing system (TTP Labtech, Melbourn, UK). Sitting drops of 400 nL were set up in Greiner plates for 672 commercially available screening solutions with a 1:1 mixture of protein complex at 10mg/mL equilibrated against 150 μL of reservoir solution. The plates were stored at 18°C in a RockImager (Formulatrix, Bedford, USA) automated imaging system to monitor crystal growth. Best crystals of *Tc*PRAC in complex with the inhibitors in 1mM solutions were obtained with a solution of 0.2 M potassium phosphate dibasic and 21% (w/v) PEG-3350. Crystals with dimensions of up to 0.1 mm x 0.1 mm x 0.05 mm appeared within one week. For data collection, the crystals were flash-cooled in liquid nitrogen using a paratone-paraffin oil mixture (50%/50%) as cryoprotectant.

X-ray diffraction data were collected on beamline PROXIMA-1 at synchrotron SOLEIL (St Aubin, France). Diffraction images were integrated with the program XDS [28–32] and crystallographic calculations were carried out with programs from the CCP4 program suite [28–32]. The structures were solved by molecular replacement using Phaser [28–32] with *Tc*PRAC in complex with PYC (pdb 1W61) [16] as a template. Refinement was done using Refmac5 [28–32] with alternating manual rebuilding in Coot [28–32]. Crystallographic data and refinement statistics are shown in Table 1.

**Table 1:** Crystallographic parameters, data and refinement statistics.

	*Tc*PRAC-BrOxoPA	*Tc*PRAC-OxoPA	*Tc*PRAC-NG-P27
***Crystal parameters***			
Space group	c2	c2	c2
Unit cell dimensions (Å)	a = 129.66, b = 90.84	a = 129.24, b = 91.33,	a = 133.23, b = 90.64,
	c = 85.92, β = 126.29°	c = 85.63, β = 126.45°	c = 85.39, β = 126.04°
***Data statistics***			
Resolution limits (Å)	42.7–1.70 (1.73–1.70)^a^	42.66–1.90 (1.94–1.90)	45.3–2.00 (2.05–2.00)^a^
No. of unique reflections	86740 (4332)	61363 (4017)	54731 (3986)
Multiplicity	3.8 (3.5)	2.6 (2.6)	3.8 (3.8)
Rmerge	0.052 (0.675)	0.077 (0.599)	0.084 (0.758)
Rpim	0.037 (0.496)	0.060 (0.480)	0.058 (0.497)
Completeness (%)	98.5 (95.8)	97.6 (99.4)	98.8 (98.0)
<I/sigma(I)>	12.0 (1.7)	8.2 (1.9)	7.8 (1.7)
CC(1/2)	0.998 (0.695)	0.995 (0.645)	0.994 (0.761)
***Refinement***			
Resolution (Å)	41.4–1.70 (1.72–1.70)	41.2–1.90 (1.92–1.90)	45.4–2.00 (2.025–2.00)
․ R value, working set	0.171 (0.328)	0.189 (0.290)	0.177 (0.254)
․ Rfree	0.203 (0.425)	0.240 (0.388)	0.230 (0.331)
No. of reflections	84997 (3050)	59988 (2116)	52236 (993)
․ Non-hydrogen atoms	5971	5939	5886
․ No. of protein residues	709	728	713
․ No. of water molecules	540	361	403
․ PO_4_ ions	-	1	1
R.m.s. deviations from ideal			
․ bond length (Å)	0.010	0.011	0.010
․ bond angles (°)	1.401	1.487	1.460
Ramachandran plot (%)			
․ Preferred regions	96.0	96.4	95.6
․ Allowed regions	3.7	3.1	3.7
․ Outliers	0.3	0.6	0.7

^a^Values in parentheses are for the highest resolution shell

### PDB deposition

The refined models and structure factors have been deposited in the Research Collaboratory for Structural Biology Protein Data Bank (hyperlink "http://www.rcsb.org/") under the following accession numbers: *Tc*PRAC-BrOxoPA (6HJF), *Tc*PRAC-OxoPA (6HJG), *Tc*PRAC-NG-P27 (6HJE).

### Synthesis

Description of the synthesis of *Tc*PRAC inhibitors is given in "General Information on Chemical Synthesis" (S1 Text), "Synthetic procedures" (S2 Text) and Refs. [33–35]. The spectral characterization of the compounds is given in the "^1^H and ^13^C spectra" (S1 Spectra).

### Conformational analysis

For conformational analysis, the atomic coordinates of the *TcPRAC* structures were limited to residues 45–394 for which the electron density could be traced in all structures. Amino acids (K152, E178, R210, P280, E281, Y294), whose side chains were not visible in all the structures, were replaced by alanine. Symmetric structures were generated by swapping the chain names (e.g. A to B and B to A) before alignment. The crystal structures, the 49 models [17], and their symmetric forms were structurally aligned by rotation and translation minimizing the root mean square distances for each set of atoms subjected to Principal Component Analysis (PCA). These sets were, respectively, the dimers, the protomers, and the amino-acids (E56, F102, L127, N128, M129, C130, G131, H132, G217, N218, F220, D269, C270, V288, F290, G291, D296, S298, C300, G301, T302, G303) defining the pocket used for virtual screening in the previous study [17].

In the description of the analysis, the protomers from the crystal structures are named according to the following scheme: ***Code-C***, or ***Lig***-***C***, with ***Code***, the PDB code, and ***Lig***, OxoPA or NG-P27, the ligand present in the structure, and ***C*** the chain, A or B of the protein. The protomers from the transitional models are designated: conf***N*C**, with ***N***, the index of the model intermediate in the transitional series, and ***C***, the chain name as indicated above.

### Calculation of pocket cavity volume

We used an in-house program to calculate cavity volumes in the pockets on a grid of voxels [36, 37]. Settings and parameters were similar to those previously reported [17], but further refinements were incorporated to delineate cavities opened to the bulk solvent. The latter was scooped out with a large rolling probe sphere of 10 Å radius with an extra erosion shell of 3.3 Å to remove the exposed part of the cavity. To discriminate the volume of the channel induced by opening from the volume delineated by the binding pocket, only cavity voxels that had one of the residues of the pocket as closest neighbor were counted.

### Parasite cultures

Epimastigote forms of CL Brener (clone F11-F5) and Y strains of *Trypanosoma cruzi* [38] constitutively expressing luciferase were maintained by weekly passage in Liver Infusion Tryptose (LIT) medium. Stock solutions (1M) of Benznidazole and NG-P27 were prepared in DMSO and subsequently diluted in LIT medium. Trypanosomal growth inhibition was determined by the evaluation of parasite number after 72h in triplicates. 10^5^ epimastigotes cultured in white microtiter plates in absence or presence of 5–1000 μM of NG-P27 were compared to results obtained with the same concentrations of Benznidazole (added once, or every day for three days) in a final volume of 200 μL. Luminescence signals emitted by live parasites in the microplate wells were quantified by the addition of 0.3 mg/mL D-luciferin (122799, Perkin Elmer, Villebon-sur-Yvette, France) in a TECAN luminometer (Infinity F200 Pro, Lyon, France). IC 50 was determined by using a non-linear fitting of the percentage of inhibition calculated after comparison to untreated controls.

## Results

### Structures of *Tc*PRAC-BrOxoPA and *Tc*PRAC-OxoPA complexes

BrOxoPA and OxoPA were co-crystallized with the enzyme. The crystallographic data and refinement statistics are shown in Table 1. The complexes crystallized in the same space group and with similar unit cell dimensions as the *Tc*PRAC/PYC complex structure reported previously (pdb 1W61) [16]. The crystallographic asymmetric unit contains two independent polypeptide chains (A, B) forming a homodimer.

**Fig 1:**
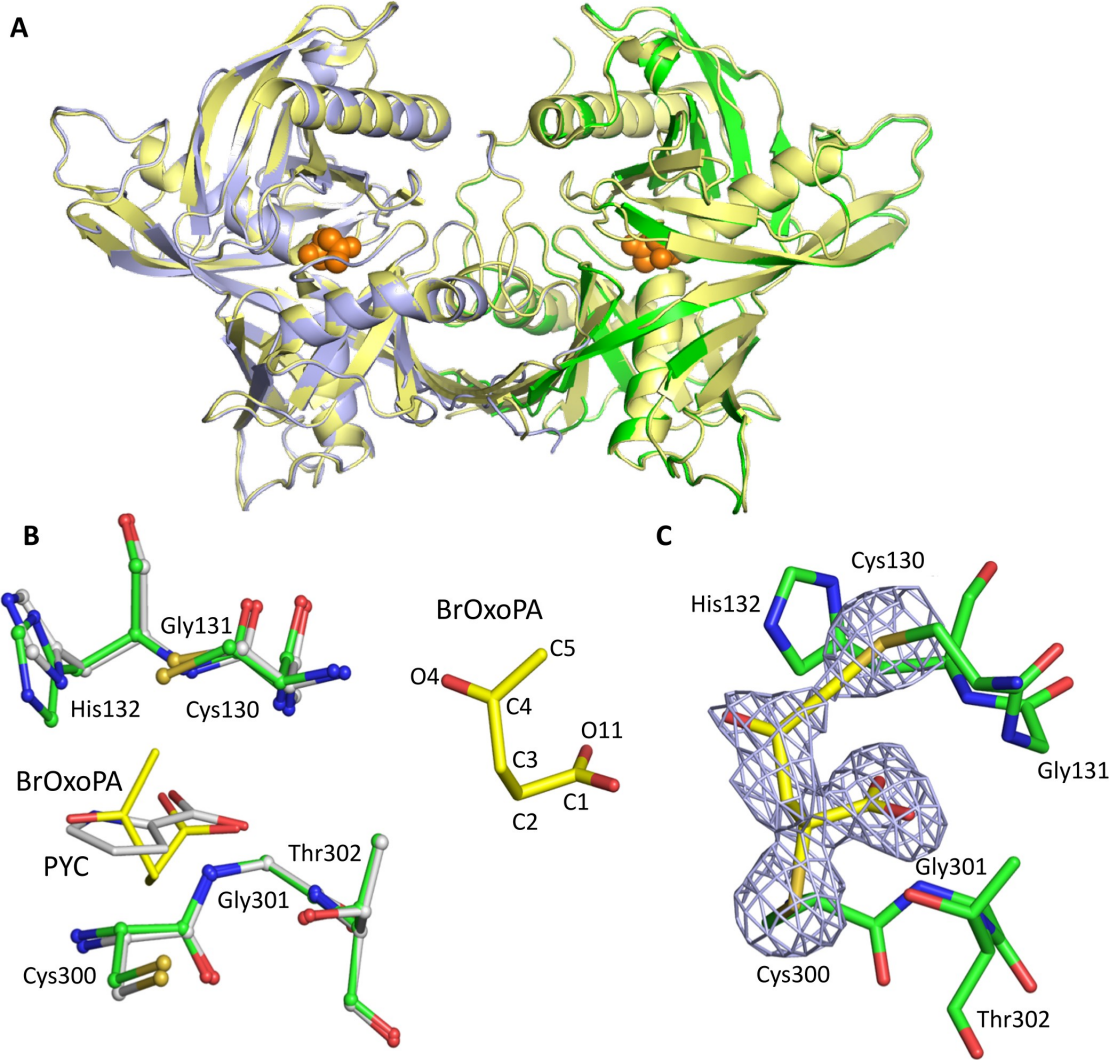
Comparison of the *Tc*PRAC structures in complex with BrOxoPA and PYC. (**A**) Superimposed *Tc*PRAC structures: the complex with PYC (yellow); *Tc*PRAC monomers in complex with BrOxoPA (green and light blue) and BrOxoPA (shown as orange spheres). (**B**) Superposition of the ligands and key residues of the catalytic site. BrOxoPA is shown in yellow and PYC in gray (**C**) 2Fo-Fc electron density omitmap contoured at 3σ of BrOxoPA after reaction, showing the covalent bonds to Cys130 and Cys300. Atom numbering is displayed on the left.

In the *Tc*PRAC-OxoPA complex, continuous electron density was seen for residues 40–394 in chain A and 45 to 398 in chain B. The overall structure closely resembles that of the enzyme in complex with PYC (1W61; Fig 1A), with an overall RMS deviation in alpha carbon positions of 0.227 Å. The position and orientation of the side chains of residues in the active site region are similar in both structures (Fig 1B). Significant electron density for the inhibitor was observed in the active site of both chains (Fig 1C). Atoms C2 and C5 of BrOxoPA are covalently bound to the sulfur atoms of Cys300 and Cys130, respectively (Fig 1C, S1 Table). These covalent interactions can explain the irreversible inhibition of *Tc*PRAC by BrOxoPA. The carboxylate moiety of the inhibitor also forms a hydrogen-bonding network with the main polypeptide chain or side chains of Gly131, His132, Gly301, and Thr302 in the active site region (Fig 1C, S1 Table). These residues are also involved in the non-covalent binding of the PYC inhibitor as previously described [16]. No electron density was observed for the bromine atom of the BrOxoPA inhibitor in difference Fourier maps, and X-ray fluorescence scans indicate the absence of bromine in crystals of the complex, suggesting that the bromine atom of the inhibitor was cleaved during the binding reaction of BrOxoPA with the enzyme.

The *Tc*PRAC-OxoPA structure presented similar global characteristics. However, comparison with the other structures is less straightforward due to greater dissymmetry between the two monomers. The impact of ligand binding on *Tc*PRAC conformation is described below and a more comprehensive analysis is provided in S3 Text. In both monomers, well defined electron density is seen for the carboxylic moieties as for BrOxoPA and PYC, but the absence of well-defined density for the ketone moiety in the OxoPA complex suggests that this part of the ligand had more relaxation freedom, and covalent interaction was not observed. These observations were confirmed by pharmacomodulations performed to optimize chemically OxoPA and BrOxoPA (see below).

### Modulations to identify novel potent and specific *Tc*PRAC covalent inhibitors

**Fig 2:**
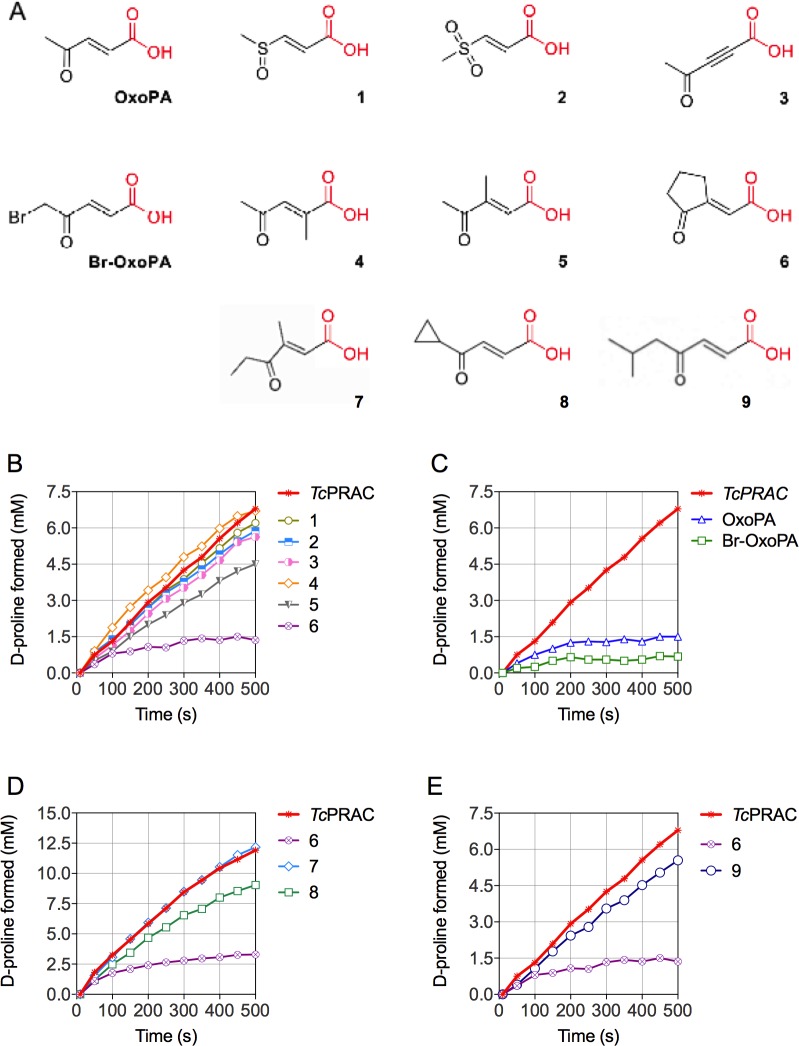
Chemical structures and activity of potential *Tc*PRAC inhibitors. (**A**) Key compounds obtained by modulation of OxoPA and BrOxoPA. (**B, C**, **E**) Kinetic curves of 40 mM L-proline racemization catalyzed by 15 μg of *Tc*PRAC in the presence of 5 μM of compounds **1** to **6** or **9 (B** and **E)** or in the presence of 5 μM of OxoPA and BrOxoPA (**C**); Kinetic curves using 100 mM L-proline racemization catalized by 10 μM of *Tc*PRAC in the presence of 10 μM of compounds **7** or **8** (**D**).

Analysis of X-ray crystallographic data of the *Tc*PRAC/BrOxoPA complex revealed two covalent bonds with the catalytic cysteine residues of *Tc*PRAC. This confirmed the high potential of the ligand and prompted us to probe the available chemical space to design a more specific covalent inhibitor. Hence, the nature of the Michael acceptor was modified and substituents were introduced at different positions of the skeleton (Fig 2).

About 60 compounds were synthesized following these principles and their ability to inhibit *Tc*PRAC was evaluated by polarimetric assay. Compounds **1** to **6** are key examples of the pharmacomodulations (Fig 2A). Their respective activities on *Tc*PRAC (Fig 2B) are compared with those of OxoPA and BrOxoPA (Fig 2C). Replacement of the ketone group with the highly electroattractive sulfoxide (**1**) or a sulfone (**2**), commonly used to design Michael acceptors [39], abolished inhibition. Differences in spatial arrangement and geometry of the tetrahedral sp3 sulfoxide/sulfone as compared to the trigonal sp2 ketone could explain this unforeseen result. Introduction of a triple bond (**3**) instead of the classical double bond Michael acceptor also abolished inhibition. Further modulations were performed on the skeleton to probe possible extension/modulation sites. Addition on the electrophilic site, C-2, (**4**) close to the Cys300 attack position, prevented inhibition. By contrast, additions at positions C-3 (**5**, **6**), C3 and C5 (**7**) or C-5 only (**8, 9**) moderately affected or maintained inhibition (Fig 2D et 2E). Compound **6** (NG-P27), bearing a five-membered ring (reminiscent of proline) was the most active derivative.

### Kinetics of *Tc*PRAC activity in the presence of inhibitors

**Fig 3:**
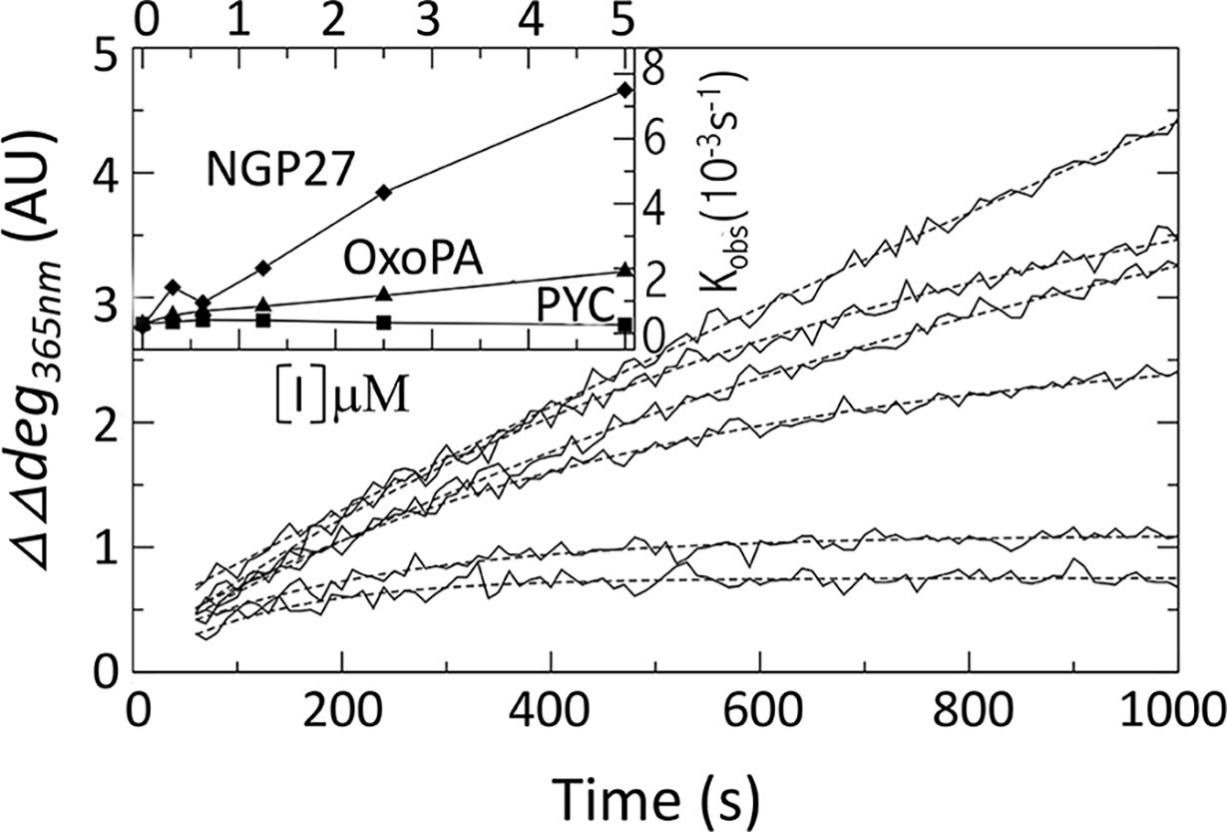
Inhibition kinetics. Main panel: Racemisation curves of 40mM of L-Proline in the presence of 5 μg of *Tc*PRAC and 0.0, 0.3125, 0.625, 1.25, 2.5 and 5 μM of NG-P27 inhibitor are reported by continuous lines from top to bottom respectively. Single exponential fits are given by underlining dashed curves. Insert: Rates of the exponential fits are given for NG-P27 (diamonds), OxoPA (triangles), and PYC (squares). Xmgrace was used to generate the graphics [http://plasma-gate.weizmann.ac.il].

Inhibition kinetics were recorded at various concentrations of PYC, OxoPA and NG-P27 (Fig 3). The kinetic curves in the absence of inhibitor were linear, validating the initial velocity approximation. Except for PYC, the asymptotes of the single exponential fit model depended on the inhibitor concentration and differed from the zero rotatory power line. Hence, the enzyme appeared to reach full inactivation during the reaction. This is typical of an irreversible inhibition mechanism and rules out a reversible competitive inhibition mechanism.

The apparent exponential kinetics constant (k_obs_) varied linearly with the concentration of OxoPA and NG-P27 as expected for an irreversible inhibition mechanism (Fig 3, inset). These results indicate that NG-P27 is a significantly more potent inhibitor than OxoPA. By contrast, the k_obs_ values were nearly zero for PYC, a reversible competitive inhibitor.

### Structural characterization of NG-P27 in the active site of *Tc*PRAC

The NG-P27 compound was co-crystallized with *Tc*PRAC in the same conditions as the BrOxoPA complex (Table 1). The overall structure of the *Tc*PRAC/NG-P27 complex and the conformation of residues in the active site displayed some deviation to the *Tc*PRAC/BrOxoPA, *Tc*PRAC/OxoPA and *Tc*PRAC/PYC complexes, but remained globally similar.

**Fig 4:**
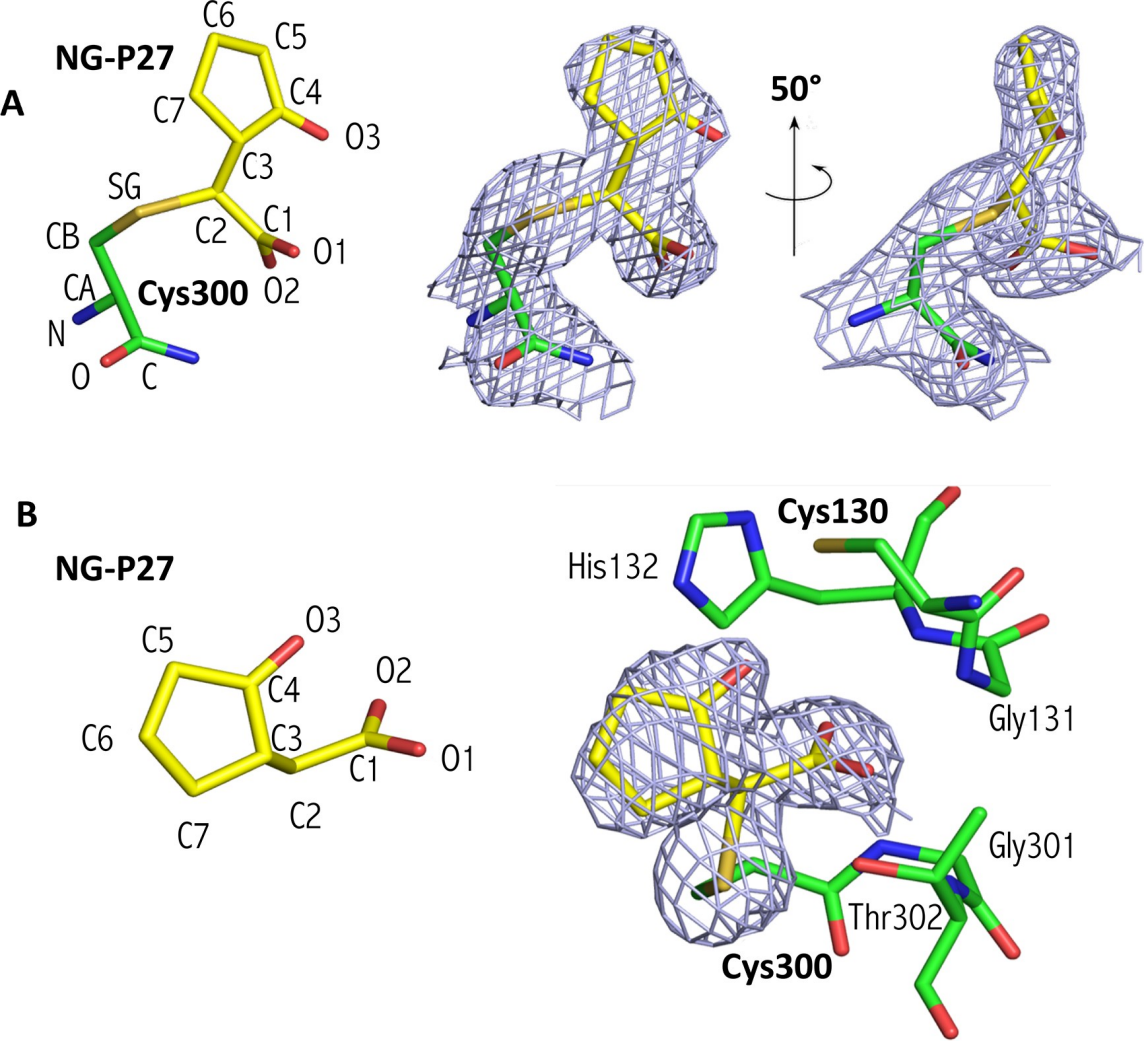
Structure of NG-P27 after reaction in the *Tc*PRAC binding site. (**A**) Left, ligand atom numbering of NG-P27 bound with the catalytic Cys300, oxygen atoms are shown in red, nitrogen in blue, and carbon in yellow for NG-P27 and green for the Cys300; Right, two views of NG-P27 bound to Cys300 with a difference electron density omitmap contoured at 3σ. (**B**) Orientation of the inhibitor and key residues in the catalytic site. The electron density map is calculated as in (**A**).

Continuous electron density was observed from residues 38–394 in chain A and 43 to 398 in chain B. Difference Fourier electron density maps indicate the presence of the inhibitor NG-P27 covalently bound to Cys300 in the active site of both polypeptide chains in the dimer. Electron density maps suggested possible multiple conformations of the cyclopentanone moiety of the inhibitor, but no attempt was made to refine alternative conformers and we describe here a single conformation of the ligand that best fits the electron density (Fig 4). The C2 atom of NG-P27 is covalently linked to the sulfur Sγ atom of the catalytic Cys300 (Fig 4, S2 Table). In both monomers the inhibitor is very tightly packed, making numerous van der Waals contacts in the active site, and a hydrogen-bonding network stabilizes the carboxylate moiety of NG-P27 as observed for the *Tc*PRAC/BrOxoPA complex (Fig 4B and S2 Table).

### Impact of ligand binding on *Tc*PRAC conformation

Ligand binding strongly affects the conformation of the protomers. For example, the two protomers of the hemi-saturated PYC-*Tc*PRAC complex (pdb: 1W62) deviate by 2.25 Å RMS on Cα positions after alignment. To gain a more comprehensive understanding of the relative motions in all the structures (5 crystal dimers, and 49 intermediate dimer models) we used Principal Component Analysis (see S3 Text for details).

### Dimers

**Fig 5:**
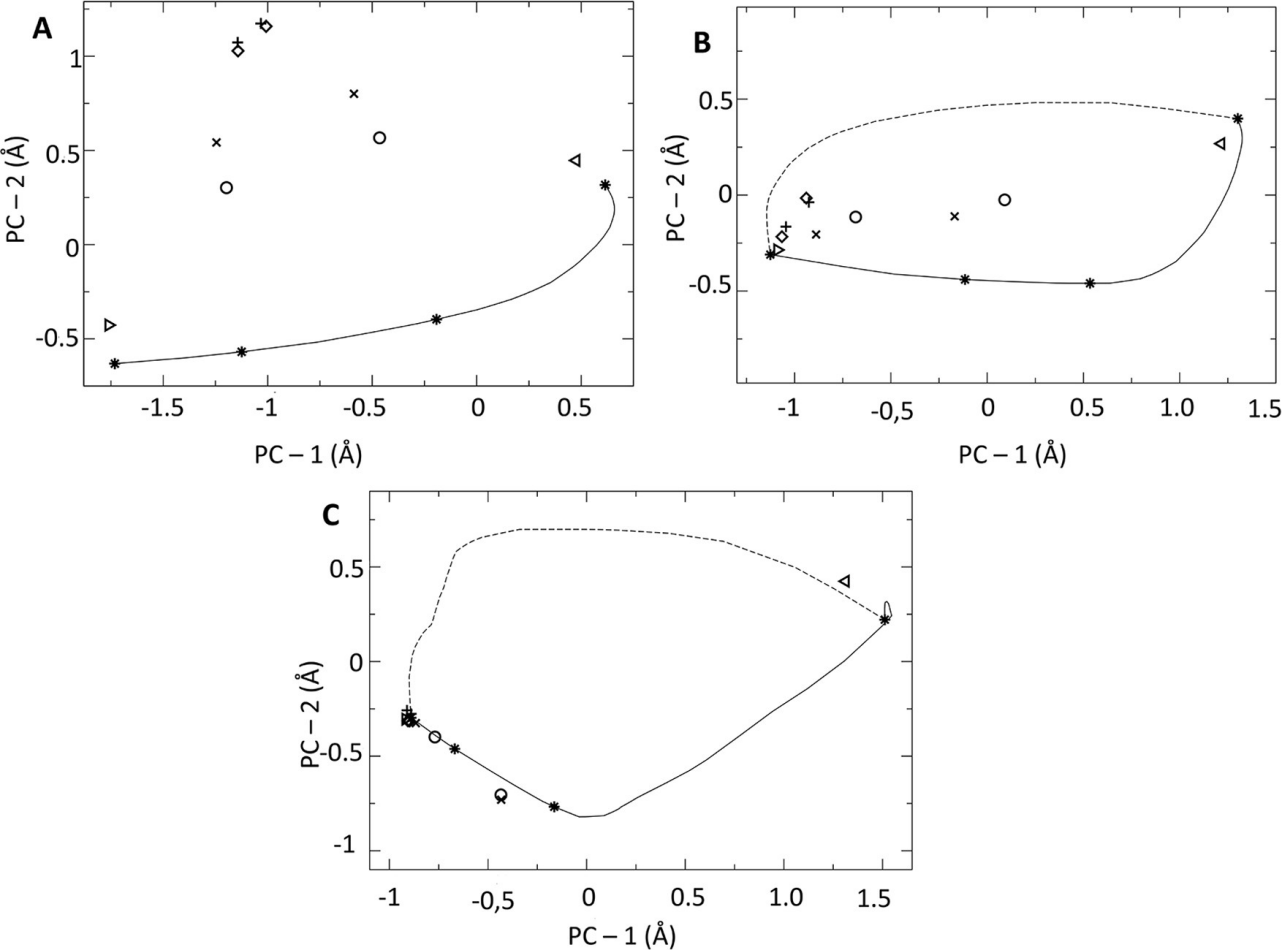
Projections on the first two Principal Components (PCs), (A) of the whole dimers coordinates and symmetric forms, (B) of chains A or B isolated, and, (C) of the binding pocket of chain A or B. Triangles pointing right/left mark 1W62/its-symmetric or chain A/B. Diamonds mark 1W61; "+", BrOxoPA complex; "x", OxoPA; and "o", NG-P27, respectively. The 49 transition path intermediates are connected by lines and conformations 1, 4, 10 and 49 used for the virtual screening are shown by "*". The 49 intermediates of chain B are connected by dashed lines.

The dimers and their symmetric forms (chains A and B swapped) roughly projected as a triangle on their first two Principal Components (Fig 5A). The saturated closed forms (1W61 and BrOxoPA) marked the top vertex. The hemisaturated 1W62 and its symmetric form constituted the base of the triangle, approximately followed by the transition models. Interestingly, OxoPA and NG-P27 displayed structures roughly midway between 1W61 and 1W62 in their direct and symmetrized forms, respectively.

#### Protomers

Analysis performed on the oriented protomers yielded projections that were globally aligned (Fig 5B). The transition models of chains A and B followed different paths. Interestingly, OxoPA-B (see naming schemes in Materials and Methods) and both chains for NG-P27 adopted intermediate conformations. Their B chains were closest to the model conformation 4 of chain A (conf4A), which led to the identification of BrOxoPA and OxoPA [17].

#### Binding site

**Fig 6:**
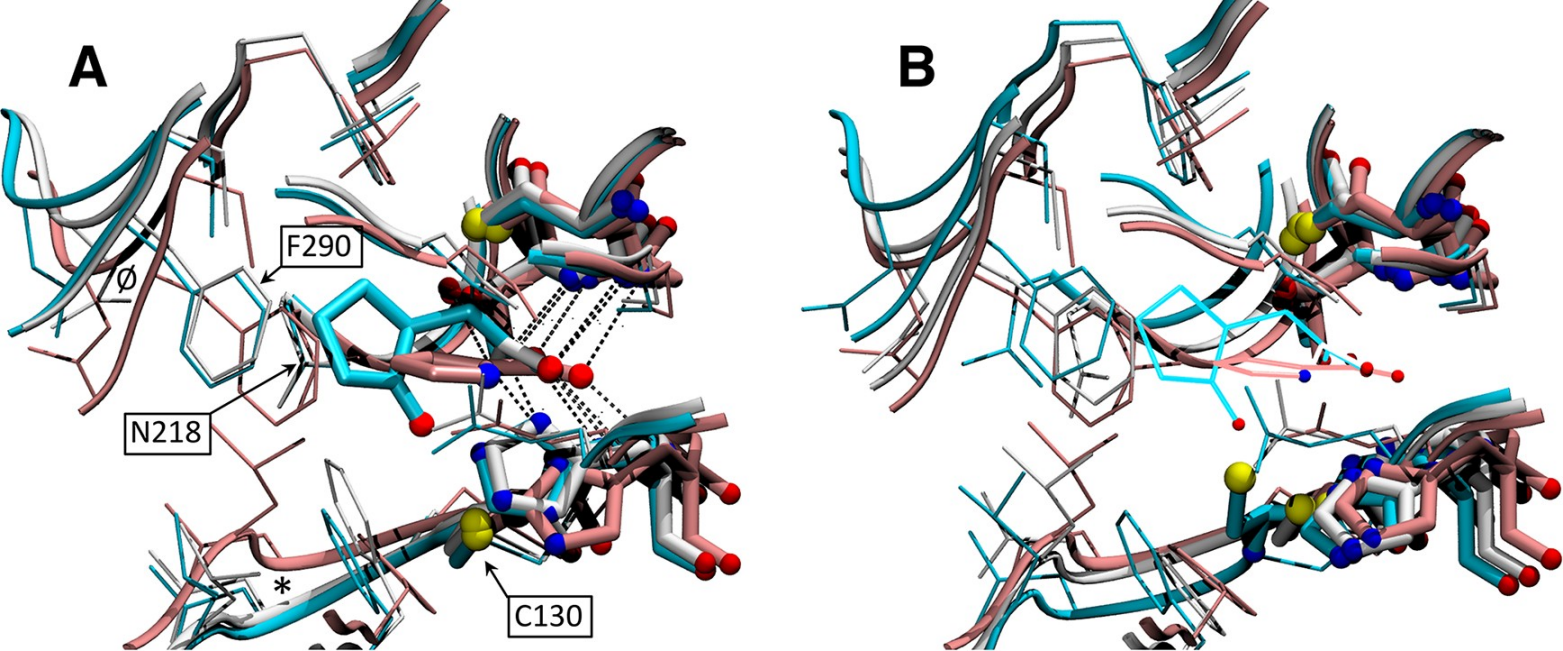
Close views of the binding sites showing conformational changes for (A) the crystal co-complexes with PYC (1W62-A, pink), OxoPA (chain B, white), and NG-P27 (chain B, cyan), and for (B) for transitional model intermediates, conf1A (pink), conf4A (white) and conf10A (cyan). Main chains are presented by ribbons, sidechains, thin sticks for amino acids of the binding site (see list in Materials and Methods). Catalytic cysteines, the ligand and residues making hydrogen bonds (dashed lines) with the ligand carboxylic moiety (C130 G131, H132, C300, G301, T302) are displayed in bolder sticks, and non carbon atoms as blue, red, and yellow spheres for nitrogen, oxygen and sulfur, respectively. The crystallographic ligands are also represented with lines in the transitional models for reference. Amino acids C130, N218, F290 are labeled, and regions 127–130 and 289–291 of the backbone are labeled with * and Ø signs.

In the analysis of the binding site, used for the virtual screening [17], the closed conformations were tightly clustered (Fig 5C). Strikingly, projections for conf1A, conf4A, and conf10A were closely aligned with those of both NG-P27-A and NG-P27-B and OxoPA-B (OxoPA-B—conf4A: 0.98 Å; NG-P27-A—conf2A: 0.82 Å (0.90 Å of conf4A); NG-P27-B—conf5A: 1.16 Å (1.17 Å of conf4A)). Projections of the two B chains were almost midway between those of models conf4A and conf10A. Fig 6 compares the structures of the binding site for intermediates along the transitional model with those of the *Tc*PRAC crystal structures when binding ligands of increasing size. PCA showed that the conformational changes observed by crystallography were globally well anticipated in the models (Fig 5C). Nonetheless some local traits were found either in the "early" model, conf4A (e.g. residues N218 or C130; Fig 6A), or in the "later" model (conf10A; e.g. residue F290, labeled backbone traces).

Analysis of the volumes delineated by the binding site (Table 2) gave similar conclusions and further usage perspectives. The complex with BrOxoPA, which formed two bonds within each site, displayed the smallest volumes. Sites in OxoPA-A and NG-P27-A had similar volumes to those found with PYC in 1W61-A/B and 1W62-A. Interestingly, in B chains, the binding sites were enlarged and had partial access to the solvent, with volumes similar to those of conf4A and conf10A of the transitional model.

**Table 2:** Volume of the binding site cavities.

^a^Volume	1W61	1W62	BrOxoPA	OxoPA	NG-P27
Chain A	73.1	72.4	57.3	69.4	81.0
Chain B	73.5	12.9 (763.4)	63.0	118.3 (330.9)	126.1 (377.6)
^b^Transition		conf1A	conf4A	conf10A	conf49A
		70.9	87.4	123.1	96.6 (864.8)

^a^The volumes delineated by the catalytic pocket only (amino-acids used in the screening) are calculated with a suite of programs developed by Desdouits et al. [36, 37] as explained in Materials and Methods and given in Å^3^. When pockets are accessible from the bulk solvent through a channel, the sum of its volume with that of the channel are given in brackets below.

^b^The pocket structures used for the virtual screening were all from chain A of the model.

In the ligand-bound forms, residues 130–132 are folded to form up to three hydrogen bonds with the ligand carboxylate moiety Cys130 positioned for nucleophilic attack. In the apo form, 1W62-B (and conf49A), this segment adopts a radically different fold. Despite the fairly open conformation of the OxoPA-B and NG-P27-B complexes, this region is well folded for interaction with the ligand (Fig 6). This difference, not apparent from the volumes (Table 2), is reflected by the difference between the two branches of the transition model in the binding pocket (Fig 5C).

### Reactivity of inhibitors probed on cysteine proteases

**Fig 7:**
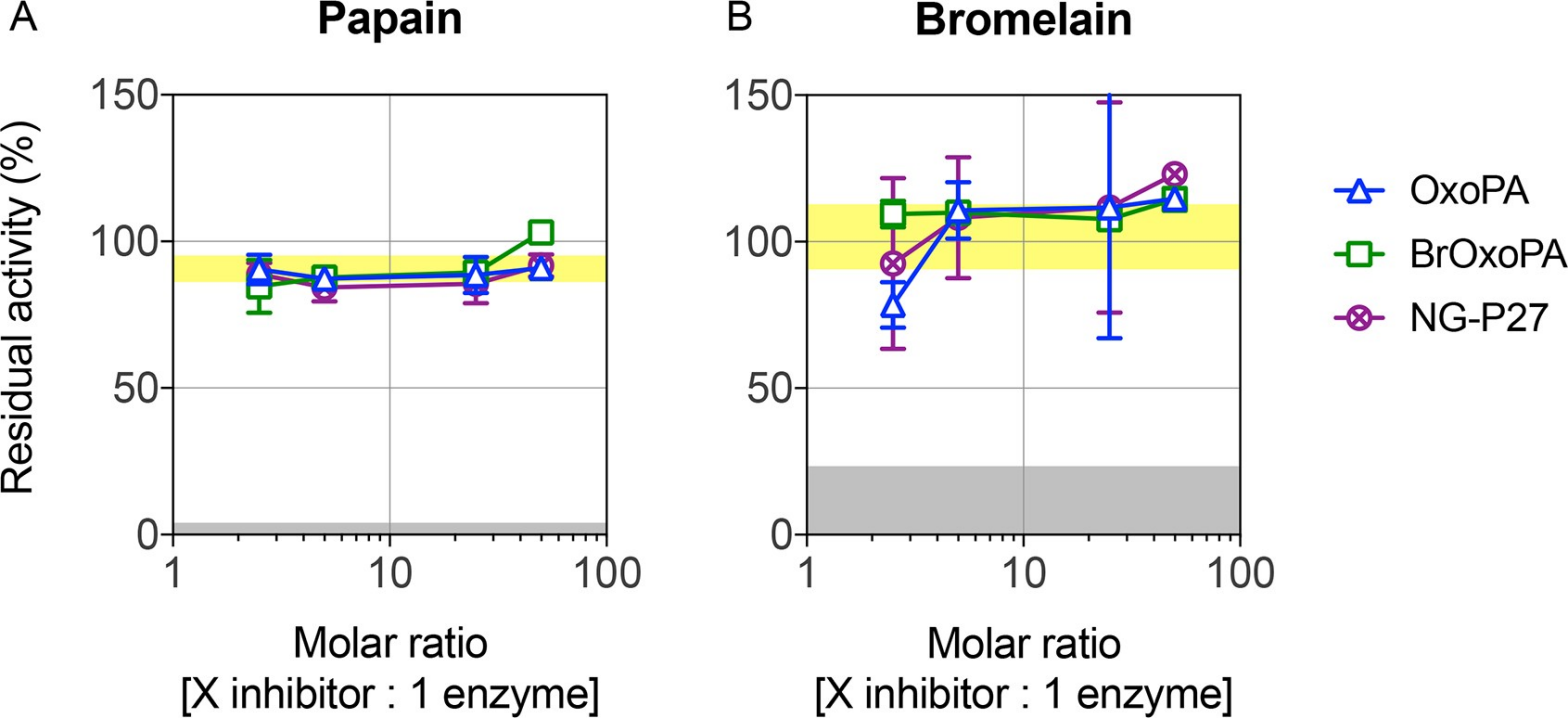
Activity of potential *Tc*PRAC inhibitors with cysteine proteases. Residual activity (%) of papain (A) and bromelain (B) after incubation with different stoichiometric ratios of OxoPA, Br-OxoPA and NG-P27. Data are expressed as mean ± SD. Residual activities after incubation with the E-64 cysteine protease inhibitor (gray hatched area) or with DMSO (light-yellow area) are shown.

One concern with Michael acceptors is their general reactivity, especially towards enzymes with reactive cysteines [40] and thus their lack of specificity. Hence, we tested the reactivity of our inhibitors towards cysteine proteases. As shown in Fig 7, the compounds did not inhibit papain or bromelain.

### Effect of NG-P27 *Tc*PRAC specific inhibitor on *T*. *cruzi* parasites

**Fig 8:**
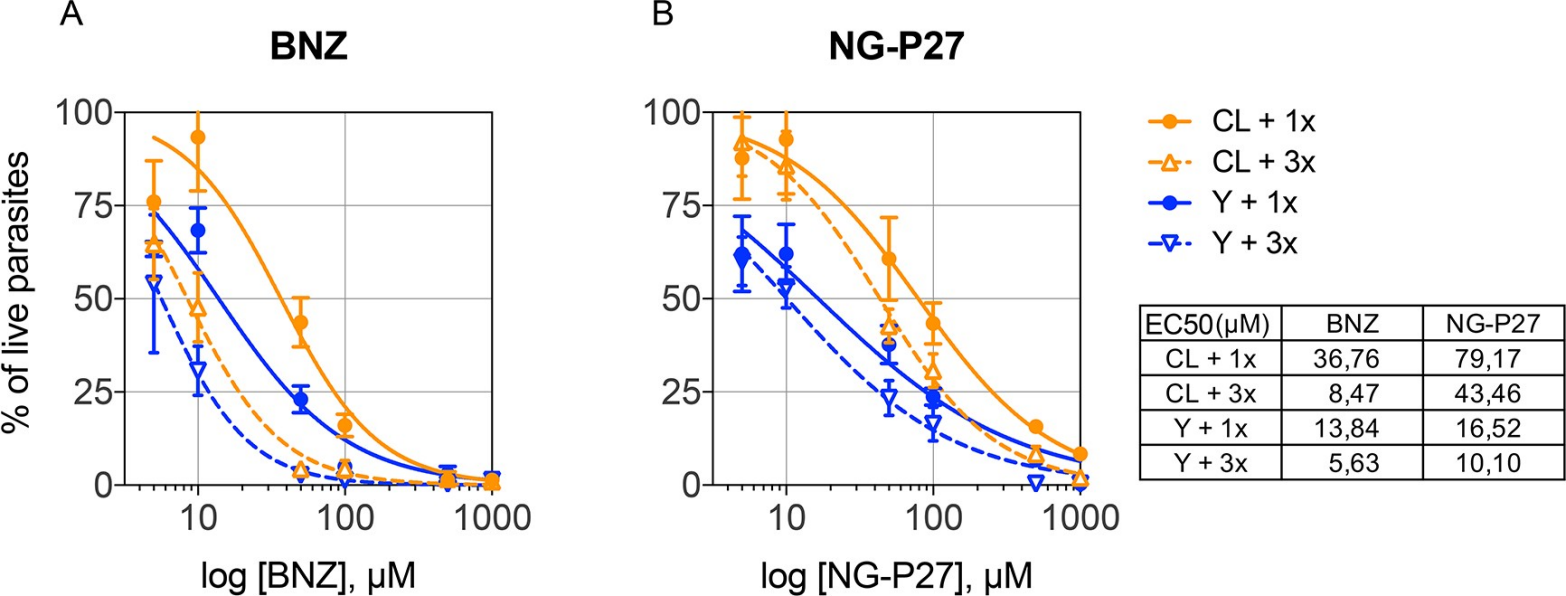
Determination of IC_50_ for epimastigote forms of *Trypanosoma cruzi*. Trypanocydal/trypanostatic effect after 72h of incubation of bioluminescent parasites of CL and Y strains with different concentrations of the reference drug benznidazole (BNZ; **A**) and the NG-P27 *Tc*PRAC inhibitor (**B**) added once (1x, complete lines) or three times, once a day for three days (3x, dashed lines).

Unlike OxoPA and BrOxoPA, NG-P27 displayed effects on parasite cultures. These effects were determined after 72h on cultures for two parasite populations and compared with the effect of Benznidazole (Fig 8). Both compounds exhibited dose-dependent trypanocydal activity for the CL and Y parasite strains. However, while multiple additions (three times) of Beznidazole increased parasite growth inhibition, no particular cumulative effect was observed after single or multiple treatments with NG-P27.

## Discussion

The search for an effective inhibitor of *Tc*PRAC was fostered by the use of structural intermediates generated between the open and closed forms of the enzyme [41]. This procedure allowed us to overcome a dead-end in the design strategy based on the PYC co-crystal structures [16], where the volume of the catalytic site was too small for a suitable chemical space search for improved inhibitors. The identification of the OxoPA and BrOxoPA inhibitors validated this method [17].

To better explore the chemical space that could be used in pharmaco-modulation, we solved the co-crystal structure of *Tc*PRAC with the OxoPA and BrOxoPA inhibitors. Structural analysis of the binding mode of the inhibitors and the induced conformational changes of the enzyme allowed us to pursue the chemical design by taking into account the chemical mechanism and protein environment both before and after reaction. This strategy led us to identify NG-P27 as a potent inhibitor of *Tc*PRAC, a key enzyme for parasite development and fate. This inhibitor could also be co-crystallized with *Tc*PRAC, revealing further details of the reaction mechanism. NG-P27 thus appeared as a promising starting point for further optimization in the search for more effective therapies against Chagas disease.

### Inhibition mechanism of *Tc*PRAC

#### Inhibitor reactive sites and design

In the BrOxoPA and NG-P27 complexes, the C2 carbon atom of the inhibitor is located approximately 2 Å from the catalytic Cys300 Sγ atom in the active site, showing the formation of a thio-ester bond (S1 and S2 Tables). The formation of a covalent bond is also implied by kinetic data for all inhibitors including OxoPA (although not observed in the crystal structure of the *Tc*Prac/OxoPA complex). Interestingly, C2 is at a position similar to that of the Cα carbon of proline, the natural substrate of the enzyme, and is at the center of a tight, highly specific network of interactions in the active site. Accordingly, this motif appeared essential in our design, as exemplified by NG-P27, and should be important for efficacy and selectivity in the future design of improved inhibitors. In the *Tc*PRAC/BrOxoPA complex, a bond is formed between Cys130 and the C5 atom of the inhibitor, as revealed in the crystal structure, and is further supported by bromine atom departure as revealed by X-ray fluorescence measurements. Indeed, alpha-bromoketones, as found in BrOxoPA, are known to be highly reactive [39]. This reactivity could result in reduced stability in biological media and lack of specificity leading to increased toxicity, both major disadvantages for drug design.

Cross-linkage has been previously reported [42] for alkylating agents such as chlorambucil, forming two bonds with its target. Furthermore, examples of double-linkage in an enzymatic catalytic site involving two of the catalytic residues have been reported with coumarine and iso-courmarine derivatives [43, 44]. Despite the promising potential of this feature, use of the alpha-bromoketones would have required important efforts to decrease its reactivity, and design in that direction was not pursued.

#### Regioselective attack at the C2 carbon of the inhibitor

**Fig 9:**
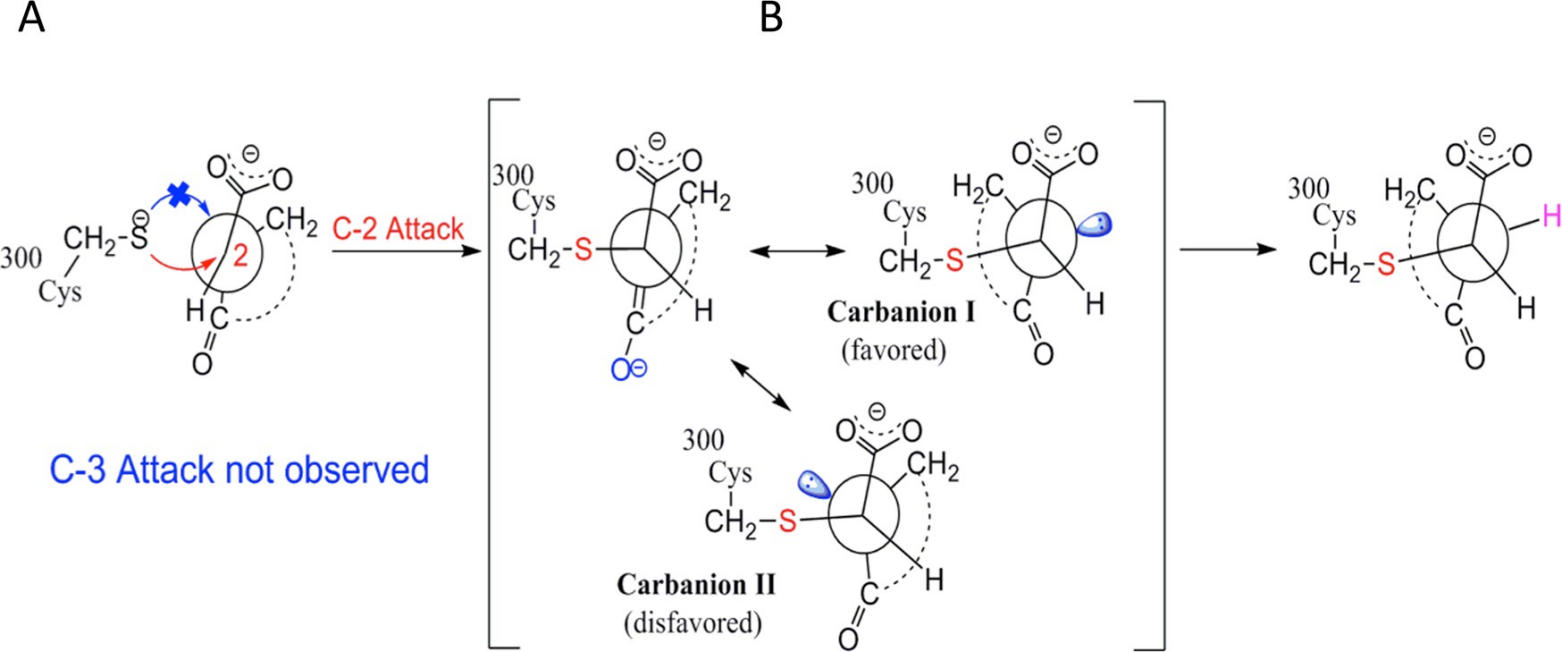
Proposed mechanism explaining (**A**) regioselectivity and (**B**) stereoselectivity in the inhibition of *Tc*PRAC by oxopentenoic derivatives.

The carbon atoms in the C2 = C3 double bond of OxoPA and its derivatives bear carboxylate and ketone substitutants respectively, allowing reaction at either position [45] (Fig 9A). Nonetheless, as revealed in the crystal structures, the reaction was regiospecific for the C2 carbon acting as a Michael acceptor, as often observed for cysteine enzymes [39] (Figs 1 and 4).

#### Stereoselectivity

**Fig 10:**
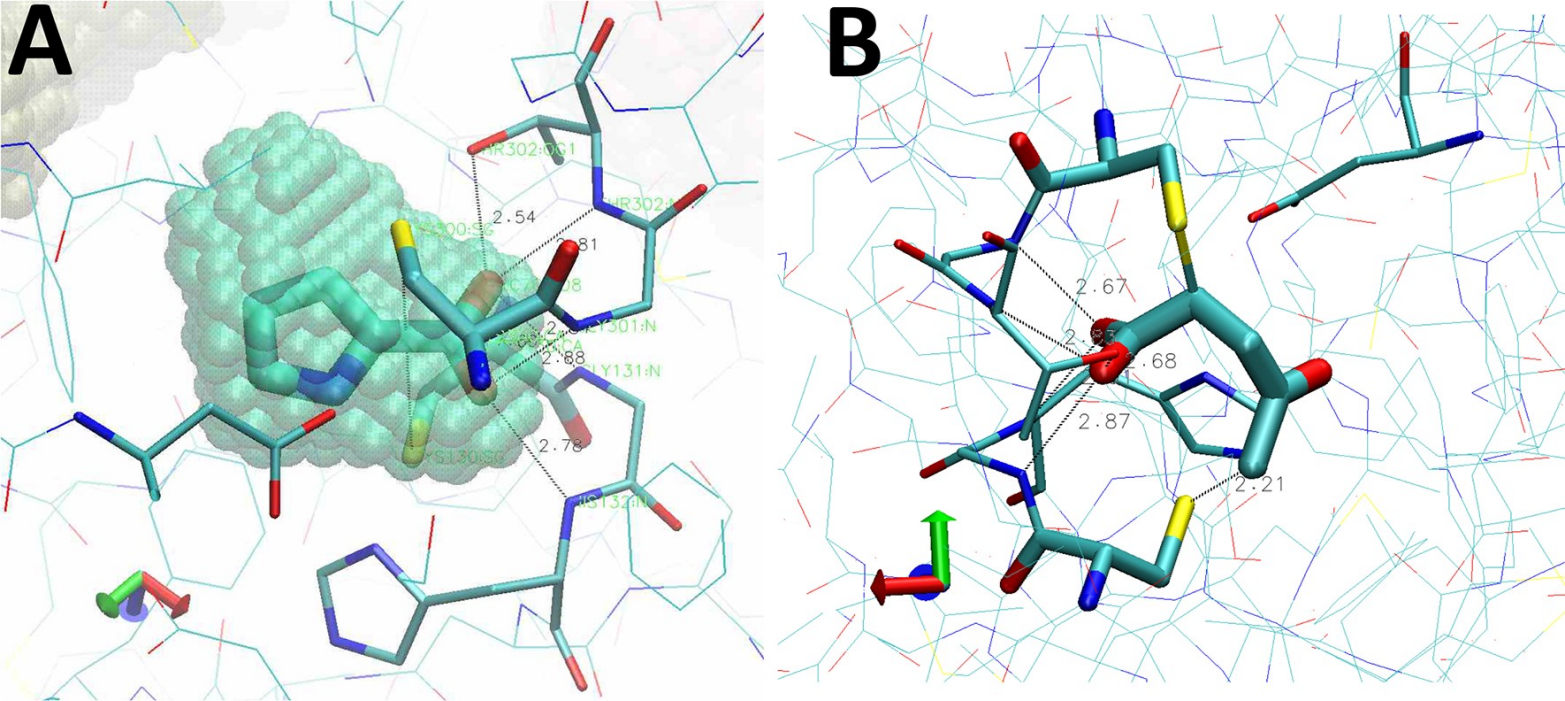
Ligand pocket in *Tc*PRAC. (**A**) Volume available as seen in the 1W62 structure; protein atoms, lines; key residues, sticks; PYC, bold sticks; and cavity volume containing PYC, mesh of transparent spheres. (**B**) BrOxoPA after reaction showing bonds with the cysteines and bending of the originally flat molecule following C2 = C3 saturation.

Attack on a Michael acceptor leads to saturation, increased flexibility, and depending on the reactant substitutions, to achiral products [46, 47] or products with one or two stereocenters [48, 49]. The crystal structure with BrOxoPA shows an enantioselective addition, 2S, at C2, while C3 remains achiral. Thus, the ketone oxygen of the inhibitor is positioned opposite to the PYC nitrogen observed in 1W62 (Fig 10A) [16], consistent with the larger available space in that direction. The BrOxoPA complex also shows that a second reaction took place, leading to a very tightly bound complex, as indicated by the cavity volume (Table 2). The saturation of C2 allows the folding of the molecule to position C5 and its bromine atom close to Cys130. The second attack released the bromine atom as reported for haloketones [39] (Fig 10B).

NG-P27, with prochiral substituents on C3, can form a second stereocenter. According to the crystallographic data, the 2S,3R configuration prevailed. Hence, the inhibitor, with C2 positioned si-face adjacent to Cys300, allowed nucleophilic addition (S) through to a mesomeric enolate intermediate (Fig 9B). The Sγ atom of Cys300 orients the inhibitor towards configuration (**I)** by repulsion of the lone pair orbitals preventing (**II**), and allowing protonation by Cys130, in the 3R configuration. This anti-process mimics the proline stereo-inversion mechanism [16]. To our knowledge, the only other reported example of a diastereoselective Michael addition in the biological field also involves an anti-process [50] (see S4 Text).

### Impact of ligand binding on *Tc*PRAC conformation

The effects of ligand binding on enzyme conformation range from the displacement of a few key residues [51] to global conformational changes [52]. The crystal structures show here a series of stable complexes in intermediate conformations representing a significant portion of the molecular mechanism (projections of protomer conformations ranging from 12 to 49% of the change on the axis between chains A and B of 1W62). This observation highlights the plasticity of the protein and its binding site. Interestingly, this flexibility does not preclude asymmetry, visible in the hemi-saturated form (1W62) and in forms bearing the same ligand in both sites (the OxoPA and NG-P27 complexes, see Fig 5A).

The protomer conformations appear to follow a mostly linear opening motion (Fig 5B). Nonetheless, projections of the dimers do not align (Fig 5A) indicating that within dimers, the protomer motions are not coupled in a linear fashion. This asymmetry can be related to the described binding anti-cooperativity of the dimers [16]. Interestingly, our transitional model [17] incorporated asymmetry compatible with the simulation force field.

In the active site, significant local reorganization such as refolding of the 130–132 loop is observed (Fig 5C). This refolding process is essential to establish interactions with the carboxylate moiety of the inhibitor and to position Cys130 for catalysis. Interestingly, in conf1A to conf10A of our transition model (Figs 5C and 6A), the 130–132 loop of chain A is folded as observed in the OxoPA and NG-P27 co-crystal structures (Fig 6B). The model for chain B was different and could represent a type of relaxations in the absence of ligand. The binding site conformation and the hydrogen bond network oriented our choice to use chain A of our transition model in our early design strategy [17].

The OxoPA and NG-P27 complex structures show that partial opening of the binding site was an appropriate assumption in our design approach. The conformation of monomer B in the OxoPA and NG-P27 complexes suggests that even larger ligands could be accommodated in the binding site (Table 2), possibly exploiting more open structures of our transitional model.

### Prospects

There is an urgent need to develop innovative drugs addressing neglected diseases, multi-drug resistance, and more broadly unmet therapeutic needs. As a new strategy for drug design, we exploit protein functional motions to model plausible structural intermediates of a therapeutic target. We show here that the *Tc*PRAC protein target adopted conformations strikingly close to those of the modeled intermediates, allowing us to design inhibitors that may lead to innovative treatments for Chagas disease. These results demonstrate how modeling protein functional motions can be exploited in a rational approach to create opportunities in drug design. This method should also be useful to complement information provided by static experimental structures for other targets involving functional molecular motions, such as GPCR, neuronal receptors, kinase involving allostery [53].

## Conclusion

Using functional intermediate models to design inhibitors proved successful with the identification of OxoPA, BrOxoPA, and finally NG-P27, a possible starting scaffold in the search for effective therapies against Chagas disease. The similarity between the binding site conformation in our models and in the crystallographic structures reported here demonstrates that computational approaches can make valuable hypotheses to exploit protein functional motions. As a perspective, this approach could also be useful to identify cryptic pockets which are now experimentally recognized as important in drug design [54].

## Supporting information

S1 Spectra ^1^H and ^13^C spectra. (DOCX)

S1 Table *Tc*PRAC / BrOxoPA intermolecular contacts. < 3.6 Å.
(DOCX)

S2 Table *Tc*PRAC / NG-P27 intermolecular contacts < 3.6 Å. (DOCX)

S1 Text General information on chemical synthesis. (DOCX)

S2 Text Synthetic procedures. (DOCX)

S3 Text Impact of ligand binding on *Tc*PRAC conformation. (DOCX)

S4 Text Inhibition mechanism of *Tc*PRAC. (DOCX)
