# Supplementary material for: Designed mono- and di-covalent inhibitors trap modeled functional motions for Trypanosoma cruzi proline racemase in crystallography
Source: PLoS Negl Trop Dis. 2018 Oct 29;12(10):e0006853. doi: 10.1371/journal.pntd.0006853 (PMC6224121; doi:10.1371/journal.pntd.0006853)
Supplement: S1 Spectra — (DOCX) [file pntd.0006853.s001.docx]

**S1 SPECTRA:** ^1^H AND ^13^C SPECTRA
