# Supplementary material for: Designed mono- and di-covalent inhibitors trap modeled functional motions for Trypanosoma cruzi proline racemase in crystallography
Source: PLoS Negl Trop Dis. 2018 Oct 29;12(10):e0006853. doi: 10.1371/journal.pntd.0006853 (PMC6224121; doi:10.1371/journal.pntd.0006853)
Supplement: S1 Table — < 3.6 Å. (DOCX) [file pntd.0006853.s002.docx]

**Table 1. Crystallographic parameters, data and refinement statistics**

|  | *Tc*PRAC-BrOxoPA | *Tc*PRAC-OxoPA | *Tc*PRAC-NG-P27 |
| --- | --- | --- | --- |
| ***Crystal parameters*** |  |  |  |
| Space group | c2 | c2 | c2 |
| Unit cell dimensions (Å) | *a*=129.66, *b*=90.84 | *a*=129.24, *b*=91.33, | *a*=133.23, *b*=90.64, |
|  | *c*=85.92, β=126.29° | *c*=85.63, β=126.45° | *c*=85.39, β=126.04° |
| ***Data statistics*** |  |  |  |
| Resolution limits (Å) | 42.7-1.70 (1.73-1.70)^a^ | 42.66-1.90 (1.94-1.90) | 45.3-2.00 (2.05-2.00)^a^ |
| No. of unique reflections | 86740 (4332) | 61363 (4017) | 54731 (3986) |
| Multiplicity | 3.8 (3.5) | 2.6 (2.6) | 3.8 (3.8) |
| Rmerge | 0.052 (0.675) | 0.077 (0.599) | 0.084 (0.758) |
| Rpim | 0.037 (0.496) | 0.060 (0.480) | 0.058 (0.497) |
| Completeness (%) | 98.5 (95.8) | 97.6 (99.4) | 98.8 (98.0) |
| <I/sigma(I)> | 12.0 (1.7) | 8.2 (1.9) | 7.8 (1.7) |
| CC(1/2) | 0.998 (0.695) | 0.995 (0.645) | 0.994 (0.761) |
| ***Refinement*** |  |  |  |
| Resolution (Å) | 41.4-1.70 (1.72-1.70) | 41.2-1.90 (1.92-1.90) | 45.4-2.00 (2.025-2.00) |
| - R value, working set | 0.171 (0.328) | 0.189 (0.290) | 0.177 (0.254) |
| - Rfree | 0.203 (0.425) | 0.240 (0.388) | 0.230 (0.331) |
| No. of reflections | 84997 (3050) | 59988 (2116) | 52236 (993) |
| - Non-hydrogen atoms | 5971 | 5939 | 5886 |
| - No. of protein residues | 709 | 728 | 713 |
| - No. of water molecules | 540 | 361 | 403 |
| - PO_4_  ions | - | 1 | 1 |
| R.m.s. deviations from ideal |  |  |  |
| - bond length (Å) | 0.010 | 0.011 | 0.010 |
| - bond angles (°) | 1.401 | 1.487 | 1.460 |
| Ramachandran plot (%) |  |  |  |
| - Preferred regions | 96.0 | 96.4 | 95.6 |
| - Allowed regions | 3.7 | 3.1 | 3.7 |
| - Outliers | 0.3 | 0.6 | 0.7 |

^a^Values in parentheses are for the highest resolution shell
