# Supplementary material for: Designed mono- and di-covalent inhibitors trap modeled functional motions for Trypanosoma cruzi proline racemase in crystallography
Source: PLoS Negl Trop Dis. 2018 Oct 29;12(10):e0006853. doi: 10.1371/journal.pntd.0006853 (PMC6224121; doi:10.1371/journal.pntd.0006853)
Supplement: S2 Table — (DOCX) [file pntd.0006853.s003.docx]

**S2 Table**. *Tc*PRAC / NG-P27 INTERMOLECULAR CONTACTS < 3.6 Å

| NG-P27 | *Tc*PRAC | Chain A | Chain B |
| --- | --- | --- | --- |
| C1  C1  C1  C1  C1  C1  O1  O1  O1  O1  O1  O1  O1  O1  O1  O1  O1  O2  O2  O2  O2  O2  O2  O2  O2  O2  C2  C2  C2  C2  C3  C3  O3  O3  O3  O3  O3  O3  C6  C7  C7 | Gly131 N  His132 CD2  Cys300 SG  Gly301 N  Thr302 N  Thr302 OG1    Cys130 C  Cys130 CA  Gly131 CA  Gly131 N  Cys300 SG  Gly301 N  Thr302 CA  Thr302 CB  Thr302 CG2  Thr302 N  Thr302 OG1    Gly131 C  Gly131 CA  Gly131 N  His132 CD2  His132 CG  His132 N  Cys300 C  Cys300 CA  Gly301 N    Asp296 OD2  Cys300 CA  Cys300 CB  Cys300 SG    Cys300 SG  Thr302 OG1    Leu127 CD1  Met129 O  Cys130 CA  Cys130 CB  Cys130 SG  His132 NE2    Phe290 CD1    Val288 CG1  Cys300 SG | 3.28  3.58  3.03  3.18  -  -    3.50  3.48  3.57  2.65 *  3.53  3.56  -  3.47  3.45  3.04 *  2.80 *    -  3.57  3.22 *  2.89  3.46  2.89 *  3.60  3.42  2.85 *    2.97  3.45  3.17  2.21^b^    2.72  3.51    3.54  3.42  3.30  3.35  3.44  -    3.59    3.23  3.06 | 3.27  -  3.09  3.25  3.56  3.47  -  -  3.32  2.69 *  -  -  3.59  3.22  3.07  2.87 *  2.61 *  3.48  3.31  3.19 *  3.06  -  2.82 *  -  -  2.98 *  3.22  3.55  3.18  2.11^b^  2.92  3.37  -  -  3.55  3.59  2.95  3.51  -  -  3.07 |

*possible hydrogen bonds; ^b^possible C-S bond
