# Supplementary material for: Designed mono- and di-covalent inhibitors trap modeled functional motions for Trypanosoma cruzi proline racemase in crystallography
Source: PLoS Negl Trop Dis. 2018 Oct 29;12(10):e0006853. doi: 10.1371/journal.pntd.0006853 (PMC6224121; doi:10.1371/journal.pntd.0006853)
Supplement: S1 Text — (DOCX) [file pntd.0006853.s004.docx]

**S1 TEXT:** GENERAL INFORMATION ON CHEMICAL SYNTHESIS

All reagents of high quality were purchased from commercial suppliers, and used without further purification. All reactions requiring anhydrous conditions were performed under an argon atmosphere using oven dried glassware. DCM and THF were distilled from CaH_2_ and Na/benzophenone, respectively. ^1^H and ^13^C NMR were recorded at 300 and 75 MHz respectively on a Bruker AM 300 spectrometer or at 500 and 125 MHz respectively on a Bruker DMX 500 WB, using CDCl_3_, DMSO (*d^6^*) or acetone (*d^6^*) (and TMS as internal standard). δ values are given in parts per million (ppm), coupling constants (*J* values) are given in Hertz (Hz), and multiplicity of signals are reported as follows: s, singlet; d, doublet; t, triplet; q, quadruplet; m, multiplet; bs, broad singlet. Thin layer chromatography was performed using precoated silica gel plate (0.2 mm thickness). All melting points are uncorrected. Infrared spectra were recorded using an Universal Attenuated Total Reflectance Accessory (UATR).
