# Supplementary material for: Designed mono- and di-covalent inhibitors trap modeled functional motions for Trypanosoma cruzi proline racemase in crystallography
Source: PLoS Negl Trop Dis. 2018 Oct 29;12(10):e0006853. doi: 10.1371/journal.pntd.0006853 (PMC6224121; doi:10.1371/journal.pntd.0006853)
Supplement: S2 Text — (DOCX) [file pntd.0006853.s005.docx]

**S2 TEXT:** SYNTHETIC PROCEDURES

(*E*)-4-Oxo-pent-2-enoic acid (**OxoPA**) [1] and (*E*)-5-Bromo-4-oxo-pent-2-enoic acid (**BrOxoPA**) [2, 3] were synthesized according to procedures in literature [1, 4, 5].

Synthesis of *Tc*PRAC inhibitors **1** and **2**.

**Scheme S1.** Synthetic route to *Tc*PRAC inhibitors **1** and **2**.

(*E*)-3-(Methylsulfinyl)propenoic acid (**1**).

Prepared according to a method described in literature [5]. White solid, mp = 145°C; ^1^H NMR (DMSO *d^6^*): δ 2.71 (3H, s), 6.28 (1H, d, *J* = 15.1 Hz), 7.94 (1H, d, *J* = 15.1 Hz), 13.01(1H, bs); ^13^C NMR (DMSO *d^6^*): δ 38.9, 124.5, 152.7, 164.6. IR-FT (UATR): 521, 934, 1003, 1237, 1283, 1625, 1692, 2508, 3042 cm^-1^. HRMS (ESI, m/z): Calcd. for C_4_H_5_O_3_S: 132.9965, found [M-H]-: 132.9966.

(*E*)-3-(Methylsulfonyl)propenoic acid (**2**).

Prepared according to a method described in literature [5]. White solid, mp = 115°C; ^1^H NMR (Acetone *d^6^*): δ 3.15 (3H, s), 6.72 (1H, d, *J* = 15.3 Hz), 7.59 (1H, d, *J* = 15.3 Hz); ^13^C NMR (Acetone *d^6^*): δ 42.2, 132.4, 144.0, 164.8. IR-FT (UATR): 486, 764, 931, 1127, 1292, 1415, 1632, 1695, 2547, 2667, 2926, 3066 cm^-1^. HRMS (ESI, m/z): Calcd. for C_4_H_5_O_4_S: 148.9914, found [M-H]-: 148.9914.

Synthesis of *Tc*PRAC inhibitor **3**.

**Scheme S2.** Synthetic route to *Tc*PRAC inhibitor **3**.

4-Oxo-pent-2-ynoic acid (**3**).

Prepared according to a method described in literature [1]. Pale yellow oil; ^1^H NMR (CDCl_3_): δ 2.44 (3H, s); ^13^C NMR (CDCl_3_): δ 32.4, 77.2, 82.0, 153.8, 182.6. HRMS (ASAP, m/z): Calcd. for C_5_H_5_O_3_: 113.0238, found [M+H]+: 113.0238.

Synthesis of *Tc*PRAC inhibitors **4**-**6**.

 **Scheme S3.** Synthetic route to *Tc*PRAC inhibitors **4**-**6**.

(*E*)-2-Methyl-4-oxopent-2-enoic acid (**4**).

Prepared according to a method described in literature [1] using orthophosphoric acid (3 mL), pyruvic acid (1.27 g, 14.0 mmol) and acetone (3 mL). White solid, mp = 90°C; ^1^H NMR (CDCl_3_): δ 2.22 (3H, d, J = 1.5 Hz), 2.34 (3H, s), 7.19 (1H, q, J = 1.5 Hz), 8.27 (1H, bs); ^13^C NMR (CDCl_3_): δ 14.1, 32.2, 134.3, 139.6, 172.8, 199.5. IR-FT (UATR): 560, 739, 803, 883, 1133, 1217, 1614, 1660, 1715, 2594, 2925 cm^-1^. HRMS (ESI, m/z): Calcd. for C_6_H_9_O_3_Na: 149.0213, found [M+Na]+: 149.0213.

(*E*)-3-Methyl-4-oxopent-2-enoic acid (**5**).

Prepared according to a method described in literature [1] using orthophosphoric acid (5 mL), glyoxylic acid (50% aq. Sol.) (7 g, 47 mmol) and butanone (10 mL). White solid, mp = 68°C; 1H NMR (CDCl_3_): δ 2.26 (3H, d, *J* = 1.4 Hz), 2.43 (3H, s), 6.61 (1H, q, *J* = 1.4 Hz), 10.49 (1H, bs); ^13^C NMR (CDCl_3_): δ 13.3, 26.2, 125.4, 152.7, 171.4, 200.0. IR-FT (UATR): 600, 877, 900, 1185, 1243, 1363, 1421, 1634, 1656, 1714, 2584, 2978 cm^-1^.

(E)-2-(2-oxocyclopentylidene)ethanoic acid (**6**)

Prepared according to a method described in literature [1] using orthophosphoric acid (5 mL), glyoxylic acid (50% aq. Sol.) (7 g, 47 mmol) and cyclopentanone (10 mL). White solid, mp = 124°C; ^1^H NMR (CDCl_3_): δ 2.04 (2H, quint., *J* = 7.6 Hz), 2.44 (2H, t, *J* = 7.8 Hz), 3.12 (2H, td, *J* = 7.4 and 2.9 Hz), 6.53 (1H, t, *J* = 2.9 Hz); ^13^C NMR (CDCl_3_): δ 19.4, 29.6, 37.8, 118.9, 153.0, 171.6, 207.2. IR-FT (UATR): 523, 888, 1167, 1245, 1425, 1661, 1682, 1729, 2575, 2639, 2902, 2952 cm^-1^. HRMS (ESI, m/z): Calcd. for C_7_H_9_O_3_: 141.0552, found [M+H]+: 141.0553.

REFERENCES

1. Kumar N, Read R. Synthesis of cyclic compounds. PCT Int Appl 2002;WO/2002/000639:<https://patentscope.wipo.int/search/fr/detail.jsf?docId=WO2002000639>.

2. Jahng Y, Kim J-I. The reaction of (E)-2,4-pentadienoic acid with aqueous bromine re-evaluation of the product. Archives of Pharmacal Research. 1989;12(3):229-30. doi: 10.1007/bf02855561.

3. Jahng Y, Kim J-I. Aza-analogue of mevinolin: Synthesis of substituted octahydroisoquinoline, the bottom half of aza-analogue of mevinolin. Archives of Pharmacal Research. 1994;17(2):104-8. doi: 10.1007/bf02974232.

4. Gouault N, Cupif J-F, Amoros M, David M. Expedient method for the solid-phase synthesis of some 4-substituted-4,5-dihydropyridazin-3(2H)-ones. Journal of the Chemical Society, Perkin Transactions 1. 2002;(20):2234-6. doi: 10.1039/B205607K.

5. Moon JT, Ha SH, Lee SH, Kwon TH, Oh CR, Kim YD, et al. Total synthesis and biological evaluation of methylgerambullone. Bioorganic & medicinal chemistry letters. 2010;20(1):52-5. Epub 2009/12/01. doi: 10.1016/j.bmcl.2009.11.040. PubMed PMID: 19945876.
