# Supplementary material for: Designed mono- and di-covalent inhibitors trap modeled functional motions for Trypanosoma cruzi proline racemase in crystallography
Source: PLoS Negl Trop Dis. 2018 Oct 29;12(10):e0006853. doi: 10.1371/journal.pntd.0006853 (PMC6224121; doi:10.1371/journal.pntd.0006853)
Supplement: S3 Text — (DOCX) [file pntd.0006853.s006.docx]

**S3 TEXT:** IMPACT OF LIGAND BINDING ON *Tc*PRAC CONFORMATION.

Ligand binding strongly affects the conformation of the protomers. In the hemi-saturated PYC-*Tc*PRAC complex (pdb: 1W62), the two superimposed protomers deviate by 2.25 Å RMS on Cα, while in the saturated form (pdb: 1W61) they only deviate by 0.407 Å. The new structures displayed variations in conformations and different level of asymmetry.

In the saturated di-covalent BrOxoPA complex, the rms deviation was was 0.50 Å. The OxoPA and NG-P27 complexes showed intermediate asymmetry, with rms deviations of 1.069Å and 0.922Å, respectively. We used Principal Component Analysis (PCA, see Materials and Methods) to extend the view to all the subunit structures (10 structures, 45 distances) and the transition model used to identify the first inhibitor (108 structures, 5778 distances).

Cumulated variance for the first 1, 2, and 3 Principal Components (PC) for the dimeric structures represented 56%, 76% and 86%, respectively. Structures projected on the first two PCs were distributed roughly on a triangle (Figure 5A). Saturated forms, (1W61) and the complex with BrOxoPA, yield one vertex; hemi-saturated *Tc*PRAC (1W62) and its symmetric form mark the other two vertices. The transition connects the latter vertices. OxoPA and NG-P27 complexes are roughly midway on each edge connecting the saturated and the two symmetric hemisaturated forms. Noticeably, the transition path deviated from the saturated form as expected from anti-cooperativity of the two catalytic sites [1].

The analysis was then focused on oriented monomers. The first 1, 2 and 3 PCs cumulated 77%, 85% and 90% of the variance. The first PC conveys as much variance as the first two PCs for dimers, and the distribution of points is roughly aligned as if one of the previous triangle edges had folded back on the saturated monomer vertex (Figure 5B). This vertex gathers closed protomers complexed with PYC, BrOxoPA and chain A of OxoPA. The transition presented a pronounced asymmetry. Interestingly, chain B of OxoPA and both chains of NG-P27 adopted intermediate conformations, some of them facing conformation 4 of chain A in the transition, which led to the identification of BrOxoPA and OxoPA as potential inhibitors [2].

Focusing on the amino acids forming the binding site (used for the virtual screening in Berneman et al. [2]), the first 1, 2 and 3 PCs represented 59%, 80% and 87% of the variance. The closed conformations are tightly clustered (Figure 5C). Pocket transition models for chain A and B differed, but conformations 1, 4, and 10 were notably aligned with that of the NG-P27 complex and chain B of the OxoPA complex. The two B chains were positioned almost midway between conformations 4 and 10.

The volumes of the cavities delineated by the above pocket were calculated with a suite of programs developed by Desdouits et al. [3]. They are reported in Table 5.

Reaction with BrOxoPA led to a shrinking of the active site of both monomers, reflecting the tight binding of the ligand upon its double reaction with the enzyme. The binding sites of chain A are more closed for OxoPA and NG-P27. Although larger than for BrOxoPA, they had similar volumes to that of the closed forms of 1W61 and 1W62. Interestingly, in chain B the sites are enlarged with partial access to the solvent. The volumes delineated by the binding pockets are similar to those of structures 4 and 10 of the transitional model [2] used to identify OxoPA and BrOxoPA. Peptide 130-132, which can form two or three hydrogen bonds with the ligand carboxylate group, has a different fold in 1W62-B and model structure 49-A, whereas it maintains its binding fold in chain B of OxoPA and NG-P27, forming hydrogen bonds and positioning the catalytic Cys130. This difference is not apparent from the volumes shown in Table 5, but leads to the difference between the two branches of the transition model in the Pocket PCA analysis (Figure 5C).

REFERENCES

1. Buschiazzo A, Goytia M, Schaeffer F, Degrave W, Shepard W, Gregoire C, et al. Crystal structure, catalytic mechanism, and mitogenic properties of Trypanosoma cruzi proline racemase. Proceedings of the National Academy of Sciences of the United States of America. 2006;103(6):1705-10. Epub 2006/02/01. doi: 10.1073/pnas.0509010103. PubMed PMID: 16446443; PubMed Central PMCID: PMCPMC1413642.

2. Berneman A, Montout L, Goyard S, Chamond N, Cosson A, d’Archivio S, et al. Combined Approaches for Drug Design Points the Way to Novel Proline Racemase Inhibitor Candidates to Fight Chagas’ Disease. PLoS ONE. 2013;8(4):e60955. doi: 10.1371/journal.pone.0060955.

3. Desdouits N, Nilges M, Blondel A. Principal Component Analysis reveals correlation of cavities evolution and functional motions in proteins. Journal of molecular graphics & modelling. 2015;55:13-24. Epub 2014/11/27. doi: 10.1016/j.jmgm.2014.10.011. PubMed PMID: 25424655.
