# Supplementary material for: Designed mono- and di-covalent inhibitors trap modeled functional motions for Trypanosoma cruzi proline racemase in crystallography
Source: PLoS Negl Trop Dis. 2018 Oct 29;12(10):e0006853. doi: 10.1371/journal.pntd.0006853 (PMC6224121; doi:10.1371/journal.pntd.0006853)
Supplement: S4 Text — (DOCX) [file pntd.0006853.s007.docx]

**S4 TEXT:** INHIBITION MECHANISM OF *Tc*PRAC

***Regioselective attack at the C2 carbon of the inhibitor***. The C2=C3 double bond of OxoPA and its derivatives are substituted by a carboxylate and a ketone group, on C2 and C3 respectively. This allows nucleophilic attack at either position [1] (Figure 9a). C2 carbon appeared at ~2 Å of catalytic Cys300 Sγ atom, typical of a thioester bond, both in BrOxoPA and NG-P27 complexes (S1 and S2 Tables). Hence, inactivation involves a Michael acceptor, as often for cysteine enzymes (mainly proteases [2]; Figure 1 and 4). The reaction is regiospecific on C2, and it is followed by protonation of the C3 carbon.

***Stereoselectivity***. Attack on a Michael acceptor leads to saturation and increased flexibility. Unsubstituted reactants yield achiral products [3, 4], while more elaborate ones can produce one or two stereocenters [5, 6]. The addition can be enantioselective if the enzyme orients the reactant.

OxoPA and BrOxoPA, have one hydrogen both on C2 and C3. Thus, C2 can become chiral upon sulfur attack, while C3 will remain achiral after protonation. Hence, crystal structures show 2S adducts at C2 of BrOxoPA, implying an enantioselective addition. This suggests that the inhibitor approaches with the ketone oxygen pointing in a similar direction as the Cβ, Cγ atoms of the proline substrate, i.e. opposite to the PYC nitrogen position in 1W62 (Figure 10A) [7]. This is consistent with the slightly larger space between Leu127 and Cys270 available in that direction.

For BrOxoPA, a second reaction took place, leading to a very tight complex as shown by the crystal structure and cavity volume (Table 2). Saturation of C2 allows rotation of the C2-C3 and C3-C4 bonds to position C5 and its bromine atom close to the Cys130 thiol for an attack releasing bromine as reported for haloketones [2] (Figure 10B).

In NG-P27, the C3 atom is bound to three prochiral carbons and can form a second stereocenter upon protonation. According to crystallographic data, the 2S,3R configuration prevailed. Hence, hydrogen bond network with the carboxylate and the wider space in the direction of Cys270 appears to orient the C2 si-face of the inhibitor close to Cys300, allowing nucleophilic addition (S) leading to a mesomeric enolate (Figure 9b). Repulsion between the lone pair orbitals and the attacking sulfur is minimized in **I**, and disfavored in **II**. In addition, protonation by Cys130 requires the lone pair positioned as in **I**, leading to 3R configuration. This anti-process mimics the proline stereo-inversion mechanism. To our knowledge, the only other reported example of a diastereoselective Michael addition in the biological field also involves an anti-process [8].

REFERENCES

1. Ekici OD, Li ZZ, Campbell AJ, James KE, Asgian JL, Mikolajczyk J, et al. Design, synthesis, and evaluation of aza-peptide Michael acceptors as selective and potent inhibitors of caspases-2, -3, -6, -7, -8, -9, and -10. Journal of medicinal chemistry. 2006;49(19):5728-49. Epub 2006/09/15. doi: 10.1021/jm0601405. PubMed PMID: 16970398.

2. Powers JC, Asgian JL, Ekici OD, James KE. Irreversible inhibitors of serine, cysteine, and threonine proteases. Chemical reviews. 2002;102(12):4639-750. Epub 2002/12/12. PubMed PMID: 12475205.

3. Huang Z, Tan L, Wang H, Liu Y, Blais S, Deng J, et al. DFG-out mode of inhibition by an irreversible type-1 inhibitor capable of overcoming gate-keeper mutations in FGF receptors. ACS chemical biology. 2015;10(1):299-309. Epub 2014/10/16. doi: 10.1021/cb500674s. PubMed PMID: 25317566; PubMed Central PMCID: PMCPMC4301177.

4. Wu H, Wang W, Liu F, Weisberg EL, Tian B, Chen Y, et al. Discovery of a potent, covalent BTK inhibitor for B-cell lymphoma. ACS chemical biology. 2014;9(5):1086-91. Epub 2014/02/22. doi: 10.1021/cb4008524. PubMed PMID: 24556163; PubMed Central PMCID: PMCPMC4027949.

5. Tan J, George S, Kusov Y, Perbandt M, Anemuller S, Mesters JR, et al. 3C protease of enterovirus 68: structure-based design of Michael acceptor inhibitors and their broad-spectrum antiviral effects against picornaviruses. Journal of virology. 2013;87(8):4339-51. Epub 2013/02/08. doi: 10.1128/jvi.01123-12. PubMed PMID: 23388726; PubMed Central PMCID: PMCPMC3624371.

6. Matthews DA, Dragovich PS, Webber SE, Fuhrman SA, Patick AK, Zalman LS, et al. Structure-assisted design of mechanism-based irreversible inhibitors of human rhinovirus 3C protease with potent antiviral activity against multiple rhinovirus serotypes. Proceedings of the National Academy of Sciences of the United States of America. 1999;96(20):11000-7. Epub 1999/09/29. PubMed PMID: 10500114; PubMed Central PMCID: PMCPMC34232.

7. Buschiazzo A, Goytia M, Schaeffer F, Degrave W, Shepard W, Gregoire C, et al. Crystal structure, catalytic mechanism, and mitogenic properties of Trypanosoma cruzi proline racemase. Proceedings of the National Academy of Sciences of the United States of America. 2006;103(6):1705-10. Epub 2006/02/01. doi: 10.1073/pnas.0509010103. PubMed PMID: 16446443; PubMed Central PMCID: PMCPMC1413642.

8. Krishnan S, Miller RM, Tian B, Mullins RD, Jacobson MP, Taunton J. Design of Reversible, Cysteine-Targeted Michael Acceptors Guided by Kinetic and Computational Analysis. Journal of the American Chemical Society. 2014;136(36):12624-30. doi: 10.1021/ja505194w.
